# *TERT* Alterations Predict Tumor Progression in *De Novo* High-Grade Meningiomas Following Adjuvant Radiotherapy

**DOI:** 10.3389/fonc.2021.747592

**Published:** 2021-10-29

**Authors:** Jiaojiao Deng, Shuchen Sun, Jiawei Chen, Daijun Wang, Haixia Cheng, Hong Chen, Qing Xie, Lingyang Hua, Ye Gong

**Affiliations:** ^1^Department of Neurosurgery, Huashan Hospital, Fudan University, Shanghai, China; ^2^Institute of Neurosurgery, Fudan University, Shanghai, China; ^3^Shanghai Key Laboratory of Brain Function Restoration and Neural Regeneration, Fudan University, Shanghai, China; ^4^Department of Neuropathology, Huashan Hospital, Fudan University, Shanghai, China; ^5^Department of Critical Care Medicine, Huashan Hospital, Fudan University, Shanghai, China

**Keywords:** high-grade meningiomas, adjuvant radiotherapy, *de novo*, *TERT* alterations, prognosis

## Abstract

**Background:**

Adjuvant radiotherapy (RT) is one of the most commonly used treatments for *de novo* high-grade meningiomas (HGMs) after surgery, but genetic determinants of clinical benefit are poorly characterized.

**Objective:**

We describe efforts to integrate clinical genomics to discover predictive biomarkers that would inform adjuvant treatment decisions in *de novo* HGMs.

**Methods:**

We undertook a retrospective analysis of 37 patients with *de novo* HGMs following RT. Clinical hybrid capture-based sequencing assay covering 184 genes was performed in all cases. Associations between tumor clinical/genomic characteristics and RT response were assessed. Overall survival (OS) and progression-free survival (PFS) curves were plotted using the Kaplan–Meier method.

**Results:**

Among the 172 HGMs from a single institution, 42 cases (37 WHO grade 2 meningiomas and five WHO grade 3 meningiomas) were identified as *de novo* HGMs following RT. Only *TERT* mutations [62.5% C228T; 25% C250T; 12.5% copy number amplification (CN amp.)] were significantly associated with tumor progression after postoperative RT (adjusted *p* = 0.003). Potential different somatic interactions between *TERT* and other tested genes were not identified. Furthermore, *TERT* alterations (*TERT*-alt) were the predictor of tumor progression (Fisher’s exact tests, *p* = 0.003) and were associated with decreased PFS (log-rank test, *p* = 0.0114) in *de novo* HGMs after RT.

**Conclusion:**

Our findings suggest that *TERT*-alt is associated with tumor progression and poor outcome of newly diagnosed HGM patients after postoperative RT.

## Introduction

Meningiomas are the most frequent tumors of the central nervous system and are generally benign (1, 2). The World Health Organization (WHO) defines three grades predictive of the risk of recurrence (3). High-grade meningiomas (HGMs) (WHO grades 2–3) are rare but aggressive tumors with considerably poorer prognosis than WHO grade 1 meningiomas (4, 5). The 5-year progression-free survival (PFS-5) and overall survival (OS) rate for HGM patients are 8%–68% and 35%–91%, respectively (4–8).

Patients with WHO grade 1 meningiomas are traditionally managed in follow-up with surveillance imaging (9). However, a standardized treatment approach to HGMs after resection has not been established (4). The benefit to survival outcomes of HGMs with adjuvant radiotherapy (RT) post-surgical resection remains unclear. Retrospective series on adjuvant RT after gross total resection led to differing results (4, 10, 11).

Meningiomas have a diverse genetic background that varies with biologic behavior (12). Alterations in the tumor suppressor gene *NF2* were the first discovered genetic etiology of meningiomas (13, 14). In *NF2* wild-type meningiomas, mutations in *TRAF7*, *KLF4*, *AKT1*, and *SMO* were noted (15–17). In addition, several mutations have been described with potential prognostic implications in HGMs (12, 14, 18). Data published recently have also shown that activating *TERT* promoter mutations, frequent inactivation of *BAP1*, deletions of *CDKN2A/B*, and mutations in *DMD* are frequent in meningiomas with malignant histological progression (18–21). These data suggest that convergent gene-expression programs may underlie HGMs, which could be leveraged to develop prognostic biomarkers.

Our previous work found that patients with *de novo* anaplastic meningiomas benefit from adjuvant RT after surgery (5, 22). However, the molecular factors associated with RT efficacy in *de novo* HGMs are largely unknown. In the present study, we describe efforts to integrate clinical genomics of 37 cases from 173 HGMs to address this issue.

## Methods

### Patient Selection

Patients were identified for study through a review of the clinical records of the Department of Neurosurgery, Huashan Hospital of Fudan University, Shanghai, China. A total of 172 HGMs (**Supplementary Table S1**) were included following study approval by the Human Subjects Institutional Review Board at Huashan Hospital, Fudan University (KY-2017-09). Clinical characteristics including age, gender, tumor location, extent of surgical resection, and outcome data were collected. In general, adjuvant radiation was recommended to both atypical and anaplastic meningioma patients, regardless of gross total resection (GTR; Simpson grades I–III) or subtotal resection (STR; Simpson grades IV–V). And the final decision was made based on the negotiation with the relatives of patients. The details of postoperative RT were described in our previous work (5). Tumor pathological subtypes were reconfirmed by at least two experienced neuropathologists. Follow-up was conducted routinely according to the guidelines of Huashan Neurosurgical Center. Written informed consent was obtained from all patients involved in our study.

A total of 172 patients with a confirmed diagnosis of HGM who met inclusion criteria (141 WHO grade 2 meningiomas and 31 WHO grade 3 meningiomas; **Supplementary Table S1**). Out of 172 cases, 87 cases received RT after surgery. Among the 87 patients, 42 (48.3%) were *de novo* meningiomas, while the remaining 45 patients presented with recurrent meningioma following prior surgical resection. And finally, 37 *de novo* meningioma samples with adequate quality of DNA concentration were included for further next-generation sequencing.

### Next-Generation Sequencing

Tumor genotyping was performed on formalin-fixed paraffin-embedded (FFPE) tumor tissue by next-generation sequencing (NGS) covering 184 genes, including common pathological relevant genes of meningiomas (**Supplementary Table S2**) (13–21, 23). Five DNA samples were excluded for sequencing due to inadequate quality of concentration. High-throughput sequencing was performed on Illumina miniseq platform by KuoRan Biomedical Technology as previously described (**Supplementary Material**) (24).

### Sanger Sequencing

The *TERT* promoter mutations were evaluated using Sanger sequencing. Genomic DNA was obtained from FFPE using the HiPure FFPE DNA Kit (Magen, D3126-03) following polymerase chain reaction-based amplification of the target region (forward primer: GGATTCGCGGGC ACAGAC; reverse primer: CAGCGCTGCCTGAAA CTC; details on PCR conditions are available upon request).

### Statistical Tests

The specific details of statistical tests are included in the figure legends. A two-tailed Fisher’s exact test was used to calculate statistical significance between different groups using a χ^2^ 2 × 2 table. Categorical variables were compared with the Fisher’s exact tests, and continuous variables with the independent-samples Student’s *t*-test (data with normal distribution) or Mann–Whitney U-test (data with skewed distribution). Continuous data were expressed as the mean ± standard deviation (SD). Overall survival (OS) and progression-free survival (PFS) curves were plotted using the Kaplan–Meier method. Statistical analysis was performed using Statistical Package for Social Sciences (SPSS, Version 20.0, Chicago, IL, USA). Data were considered to be significant when *p* < 0.05.

## Results

### Prevalence of Somatic Alterations in 37 *De Novo* High-Grade Meningiomas

**Figure 1:**
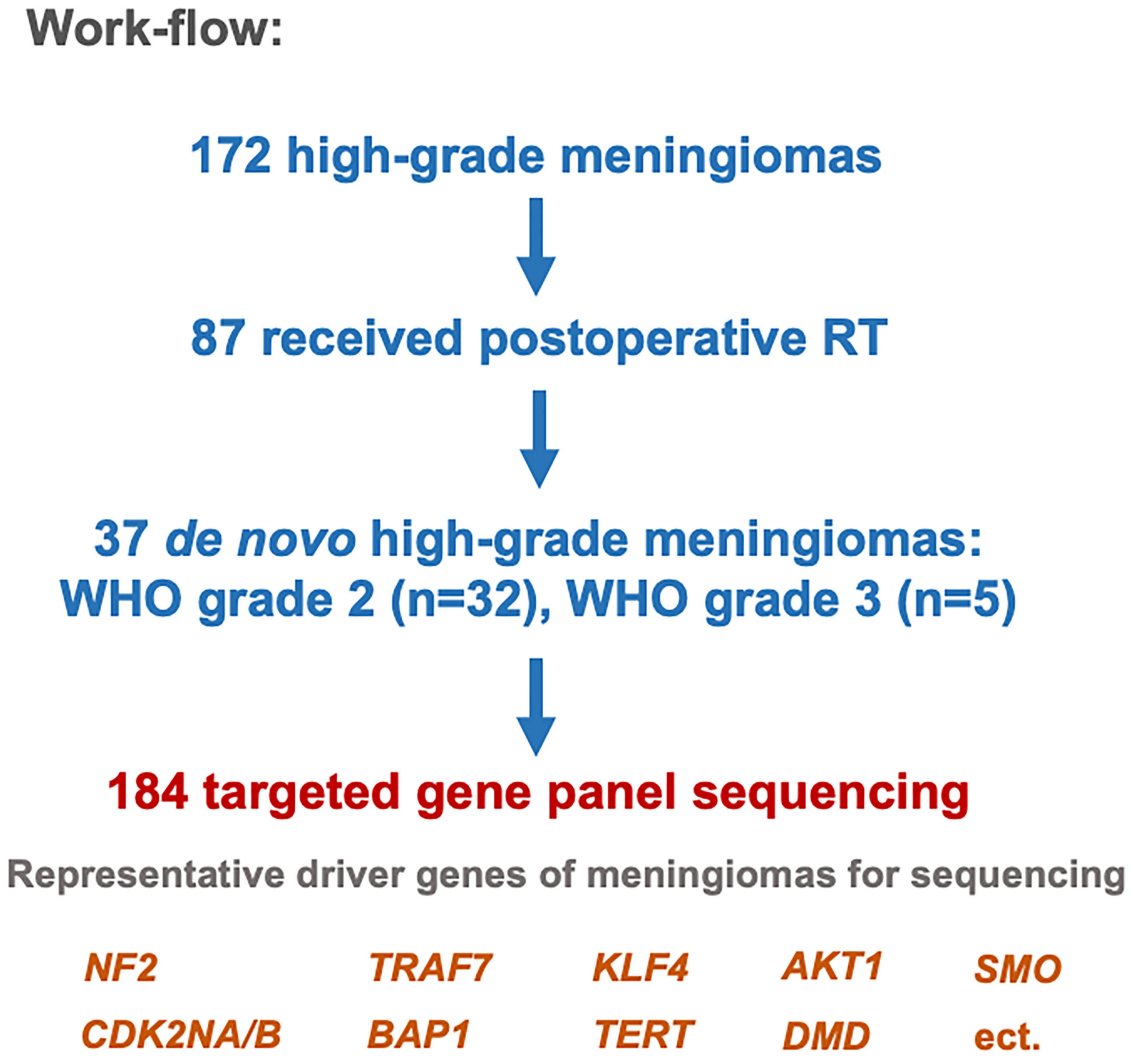
Study design.

We identified 172 patients with a confirmed diagnosis of HGM who met inclusion criteria (141 WHO grade 2 meningiomas and 31 WHO grade 3 meningiomas; **Supplementary Table S1**). Out of 172 cases, 87 cases received RT after surgery, including external beam radiation therapy (EBRT), stereotactic radiosurgery (SRS), and cyber knife (CK). Among the 87 patients, 42 (48.3%) were *de novo* meningiomas while the remaining 45 patients presented with recurrent meningioma following prior surgical resection. Five of the 42 (11.9%) meningiomas were excluded for sequencing due to inadequate quality of DNA concentration. Thus, tumor genotyping covering 184 genes was performed on the 37 *de novo* meningioma cases (**Supplementary Tables S2****,** **S3**). In total of the 37 cases, 23 males (62%) and 14 females (38%) with a median age of 45 years (range: 34–73) harboring 31 atypical (83.8%), five anaplastic (13.5%), and one atypical/chordoid coexisting (2.7%) meningiomas were included. Tumors were located at the convexity (n = 17, 45.9%), the falx/parasagittal (n = 13, 35.1%), the skull base (n = 5, 13.5%), or in other locations (n = 2, 5.5%). Among these patients, 28 (75.7%) were treated with EBRT, six (16.2%) were treated with SRS, and three cases were treated with CK (8.1%) (**Figure 1**).

**Figure 2:**
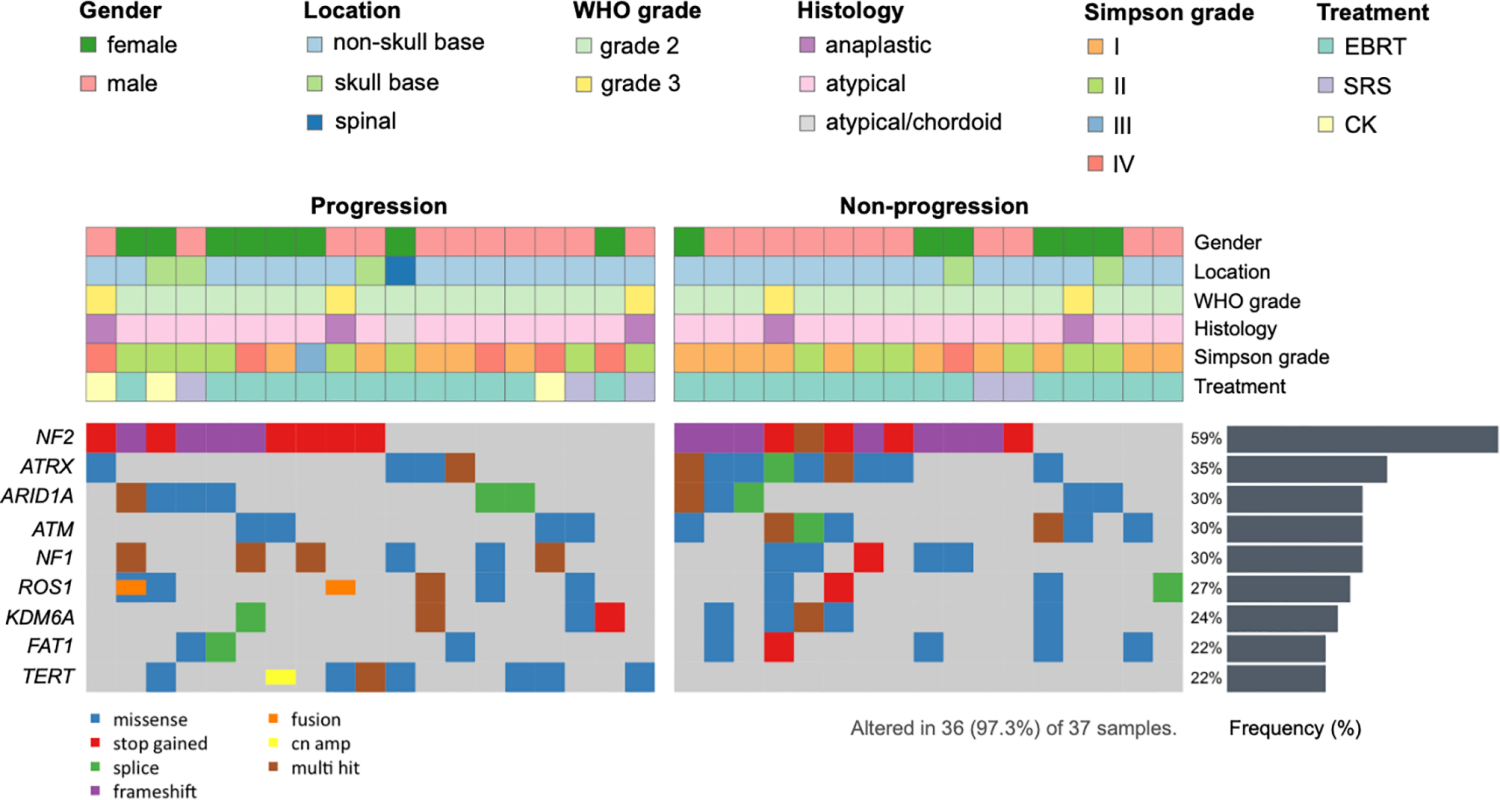
Recurrent mutations in *de novo* high-grade meningioma (HGM) patients with and without progression after adjuvant radiotherapy (RT). Top 9 genes with most frequent mutations are depicted. Each column corresponds to a single patient. The colors of bars are indicative of the type of mutation, with gray indicating wild type. Barplot at the top of the figure represents the status a patient has. The vertical plot on the right of the figure represents the frequency of mutations in each gene in a decreasing manner.

In order to identify tumor mutations associated with efficacy of postoperative RT, we determined the association between recurrent mutations and tumor progression in the cohort of 37 patients (**Figure 1**). Of the 37 *de novo* HGMs following RT, 19 cases (51.4%) had tumor progression. Progression individuals was defined as tumor regrowth within the radiation field based on the criterion of Response Assessment in Neuro-Oncology Working Group (RANO) radiologic criteria for meningiomas (25). Part of the genomic mutational landscape of 37 patients with *de novo* meningioma is displayed in **Figure 2**. Nine mutations met our predetermined recurrence frequency threshold of >20% (**Figure 2** and **Supplementary Table S2**). Consistent with previous studies, high mutational rates of *NF2* (n = 22; 59%) were discovered in this cohort. Additional common pathological relevant genes of meningiomas, including *AKT1* (n = 3; 8%), *CDKN2A* (n = 2; 5%), *SMO* (n = 0; 0%), *SUFU* (n = 0; 0%), *POLR2A* (n = 6; 16%), *TRAF7* (n = 1; 3%), and *SMARCB1* (n = 2; 5%), were observed as well (**Supplementary Table S2**). Besides, the most frequently altered genes including *ATRX* (n = 13; 35%), *ARID1A* (n = 11; 30%), *ATM* (n = 11; 30%), *NF1* (n = 11; 30%), *ROS1* (n = 10; 27%), *KDM6A* (n = 9; 24%), *FAT1* (n = 8; 22%), and *TERT* (n = 8; 22%) were observed (**Figure 2**).

### *TERT* Mutations Predict Tumor Progression of *De Novo* High-Grade Meningiomas Following Radiotherapy

**Figure 3:**
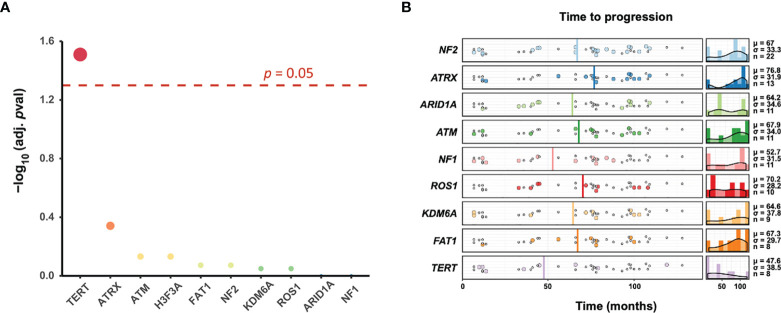
*TERT* mutations predict tumor progression of *de novo* high-grade meningiomas (HGMs) following radiotherapy (RT). **(A)** Association of recurrent mutations with tumor progression. Fisher’s test was utilized to detect differentially mutated genes on top 9 most frequent mutation genes between two cohorts (progression *vs.* non-progression). The point size in dotplot corresponds to the -log10(adj. *p*-val) value, together with the red color indicates the higher -log10(adj. *p*-val) value, and blue indicates the lower value. Horizontal dash line marked the *p*-value 0.05. **(B)** Timing of tumor progression. Shown is the time to progression (colored dots) or last progression-free scan (gray dots) for top 9 most frequent mutation genes in months. The average progression time was depicted in vertical line. The average time to recurrence of *TERT* mutant meningiomas was less than other tumors (Wilcoxon rank sum test with continuity correction, W = 491, *p* = 0.0864). Density plot of each subgroup’s progression is shown on the right, along with the mean (μ), standard deviation (σ), and number of progression (n).

Strikingly, only *TERT* mutations were significantly associated with tumor progression (n = 8, adjusted *p* = 0.031), and all these mutations were present in tumors that progressed after RT (**Figure 3A**). Of the *TERT* mutant cases, 87.5% (7/8) presented with *TERT* promoter mutations (62.5% C228T variant and 25% C250T variant; **Supplementary Figure S1**), and 12.5% (1/8) harbored copy number amplification (CN amp.). As many tumor driver genes are co-occurring or show strong exclusiveness in their mutation pattern, we next explored the potential different somatic interactions in the cohort. None of the gene mutations presents co-occurring or mutually exclusive in HGM cases (**Supplementary Figure S2**). Thus, *TERT* mutations appear to be the dominant cause of tumor progression among mutations in this cohort. Furthermore, we found that *TERT* mutation status (*p* = 0.003), as well as extent of resection (EOR) (*p* < 0.001), was significantly associated with tumor progression by Fisher’s exact tests (**Table 1**). Neither tumor location (*p* = 1.00) nor WHO grade (*p* = 1.00) predicted tumor progression after postoperative RT. After adjustment for multiple comparisons, these associations were not significant. Importantly, *TERT* mutation cases were more likely to have unfavorable time to recurrence (TTR) over the entire cohort, with a mean observation time of 47.6 months (**Figure 3B**). However, no significant differences in the average time to recurrence between *TERT* mutant and wild-type cases were observed (Wilcoxon rank sum test with continuity correction W = 491, *p* = 0.0864).

**Table 1:** Analysis of progression factors in *de novo* HGM patients after RT.

Feature		Progression	Non-progression	*p*-value
**Patients, n (%)**		19 (51.35)	18 (48.65)	
**Age, n (%)**				
	<65	13 (68.42)	14 (77.78)	0.71
	≥65	6 (31.58)	4 (22.22)	
**Gender, n (%)**				
	Male	11 (57.89)	12 (66.67)	0.74
	Female	8 (42.11)	6 (33.33)	
**Location, n (%)**				
	Skull base	3 (15.79)	2 (11.11)	1.00
	Non-skull base	16 (84.21)	16 (88.89)	
**WHO grade, n (%)**				
	2	16 (84.21)	16 (88.89)	1.00
	3	3 (15.79)	2 (11.11)	
**EOR, n (%)**				
	GTR	14 (73.68)	1 (5.56)	<0.001**
	STR	5 (26.32)	17 (94.44)	
***TERT* status, n (%)**				
	*TERT* (+)	8 (42.11)	0 (0)	0.003*
	*TERT* (-)	11 (57.89)	18 (100)	

GTR, gross total resection; HGM, high-grade meningioma; RT, radiotherapy; STR, subtotal resection; EOR, extent of resection.

*p < 0.05 and **p < 0.001 considered statistically significant.

Meningioma with *TERT* alterations, regardless of WHO grades or pathological subtypes, had a highly significant risk of recurrence (26). To exclude the disruption to RT efficacy might be caused by *TERT*-related malignant biological behavior, we thus performed analysis on newly diagnosed HGMs depending on *TERT* alterations only in progression group. Of the 19 cases in the progression group, mitotic index (ki-67%) depending on *TERT* alterations did not show any significantly difference (unpaired t-test, *p* = 0.051; **Supplementary Figure S3A**). Additionally, *TERT* alterations of *de novo* HGMs had no predictive effect on tumor recurrence in progression group following postoperative RT (*p* = 0.074 with log-rank test; **Supplementary Figure S3B**).

### *TERT* Mutations Were Associated With Decreased Progression-Free Survival and Overall Survival in *De Novo* High-Grade Meningiomas After Radiotherapy

**Figure 4:**
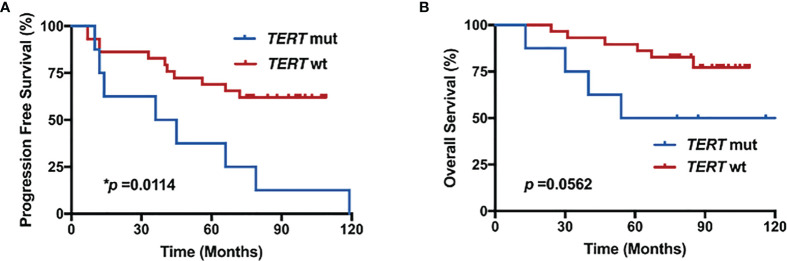
*TERT* mutations were associated with decreased progression-free survival (PFS) and overall survival (OS) in *de novo* high-grade meningiomas (HGMs) after radiotherapy (RT). Kaplan–Meier curves for **(A)** PFS and **(B)** OS in *de novo* HGMs with or without *TERT* mutation following RT.

With these findings, we next analyzed the effect of *TERT* mutation status on PFS in patients with *de novo* HGMs after RT. The primary endpoint of PFS, defined as time from surgery to date of progression, was assessed on the basis of progression of meningioma after initial surgery on imaging follow-up. The median PFS of 75 months (range: 7–109 months) was observed for the entire cohort. Subgroup analysis revealed a median PFS of 25 months (range: 10–79 months) in *TERT* mutant group and 77 months (range: 7–109 months) in *TERT* wild-type group. *TERT* mutants were significantly associated with decreased PFS in *de novo* HGM cases that underwent postoperative RT (*p* = 0.0114 with log-rank test; **Figure 4A**).

The relationship between *TERT* mutations and OS was analyzed using the log-rank test (**Figure 4B**). The median OS of 84 months (range: 13–123 months) was observed for the entire cohort. Subgroup analysis revealed a median OS of 66 months (range: 13–123 months) in *TERT* mutant group and 85 months (range: 24–109 months) in *TERT* wild-type group. Patients with *de novo* HGMs after RT harboring *TERT* mutations had worse OS (*p* = 0.0562; **Figure 4B**).

## Discussion

In this retrospective study, we investigated genetic predictors that might inform the potential progressive risk in *de novo* HGMs after postoperative RT. We found *TERT*-alt in HGMs to be a significant predictor of tumor progression compared to *TERT* wild-type cases. Although a merely descriptive finding, our data have potential implications for the clinical management of patients with *TERT*-alt *de novo* HGMs.

HGMs are rare but aggressive tumors with considerably poorer prognosis than lower-grade meningiomas. Adjuvant RT is the only nonsurgical standard of care treatment option for these tumors (9). However, radiotherapeutic options for meningioma are diverse, and there are no randomized trials to identify individuals who are more likely to benefit from adjuvant RT. Indeed, until recently, the evidence supporting postoperative RT for meningioma, especially HGMs, was largely limited. Moreover, no molecular prognostic markers have yet been established for new diagnosed HGMs following RT. To our knowledge, this study is the first to investigate predictive biomarkers that would inform tumor progression in *de novo* HGMs after postoperative RT.

Leveraging the next-generation sequencing techniques led to advances in description of the mutational landscape of meningiomas (15–17). In line with the previous findings, *NF2*-mutant meningiomas represent the largest percentage (59%) of cases in our study (14, 15). In large-scale genomic studies of meningioma, HGMs were in some studies exclusively related to pathogenic variants in *NF2*, associated with mutations in the *TERT* promoter (27). In our study, high mutational rates of *ATRX*, *ARID1A*, *ATM*, *NF1*, *ROS1*, *KDM6A*, *FAT1*, and *TERT* were observed, indicating that these frequently altered genes might play a role in HGMs. Of note, other common pathological relevant genes of meningiomas, including *AKT1* (n = 3; 8%), *CDKN2A* (n = 2; 5%), *SMO* (n = 0; 0%), *SUFU* (n = 0; 0%), *POLR2A* (n = 6; 16%), *TRAF7* (n = 1; 3%), and *SMARCB1* (n = 2; 5%), were detected as well. However, we did not observe previously described mutational rates of some of these genes due to limited cases.

Among the 184 sequenced genes, only *TERT* alterations were significantly associated with tumor progression (n = 8, adjusted *p* = 0.031), and all these mutations were present in tumors that progressed after RT. *TERT*-alt comprise, but are not limited to, promoter mutations, gene translocations, and DNA amplifications (28). We found 87.5% (7/8) of the *TERT* mutant cases presented with *TERT* promoter mutations (62.5% C228T variant and 25% C250T variant). As reported, the most common alterations occur in specific “hotspots” of the promoter (*TERT*p) region known as C228T and C250T (29). These C>T transition mutations lead to maintenance of the telomere length, as binding of E-twenty six (ETS)-transcription factors is involved in the upregulation of TERT expression (28, 30).

*TERT* alterations, specifically *TERT* promoter mutations, have been identified in a subset of HGMs with progression from low-grade meningioma (31–34). In the present study, we found that *TERT*-alt was associated with decreased PFS and OS in *de novo* HGMs after RT. Besides, our data have shown decreased time to progression among *TERT*-alt *de novo* HGMs as well. *TERT* gene is transcriptionally inactive in most non-neoplastic cells (28). *TERT* gene alterations (*TERT*-alt) may enforce cell immortalization by counteracting telomere shortening, thus promoting growth (28). Several studies have provided evidence that *TERT*-alt mutations are associated with rapid recurrence and malignant progression in meningioma (26, 31, 35). In addition, *TERT*p meningiomas have been found to have a worse PFS and OS, though not many cases have been reported (18). Activating *TERT* gene mutations in the upstream promoter allows overexpression of this enzyme and is responsible for immortalization of tumor cells in many cancers (12). Thus, we have excluded that the disruption to RT efficacy might be caused by *TERT*-related malignant biological behavior in this study. As results, the *TERT*-alt status and progressive variables did not show any relevance in progression group.

The Simpson grade of EOR has long been used to guide clinical expectations after resection of meningiomas (9); our results support the relevance of EOR in recurrence of HGMs as well (*p* < 0.001). The literature widely recognizes the role that EOR plays in determining HGM prognosis (36, 37). However, Cox regression analysis failed to identify any factor with significant association with the progression of *de novo* HGMs following RT. Thus, a larger cohort or multicenter clinical trial is needed to investigate the effect of RT in this subgroup.

In summary, our data identified *TERT* alterations, especially *TERT*p mutation, are associated with tumor progression and poor outcome of newly diagnosed HGM patients after postoperative RT. Several limitations of this study warrant consideration. Firstly, our findings on a discovery series were not substantiated by any independent validation series due to the limited available samples. Prospective studies are clearly needed to validate *TERT*-alt status of radiation response in *de novo* HGMs. Another important limitation in this study is its observational nature, which could have led to selection bias. It would be useful to repeat these analyses in cohorts from other institutions in the future.

## Conclusion

In summary, examining a cohort of *de novo* HGMs following adjuvant RT, we find *TERT* alteration to be strongly associated with tumor progression and poor outcome of HGM patients included in this study.

## Data Availability Statement

The datasets presented in this study can be found in online repositories. The names of the repository/repositories and accession number(s) can be found below: NCBI [accession: PRJNA753598].

## Ethics Statement

The studies involving human participants were reviewed and approved by the Human Subjects Institutional Review Board at Huashan Hospital, Fudan University. The patients/participants provided their written informed consent to participate in this study. Written informed consent was obtained from the individuals for the publication of any potentially identifiable images or data included in this article.

## Author Contributions

JD, LH, and YG, study design. JD, SS, JC, DW, and QX, clinical data. JD, article preparation. HaC and HoC, tumor pathological subtypes reconfirmed and Sanger sequencing. All authors contributed to the article and approved the submitted version.

## Funding

This study was supported by grants from the National Key R&D Program of China (2018YFC1312600 and 2018YFC1312604 to YG) and the National Natural Science Foundation of China (82072788 and 81772674 to YG).

## Conflict of Interest

The authors declare that the research was conducted in the absence of any commercial or financial relationships that could be construed as a potential conflict of interest.

## Publisher’s Note

All claims expressed in this article are solely those of the authors and do not necessarily represent those of their affiliated organizations, or those of the publisher, the editors and the reviewers. Any product that may be evaluated in this article, or claim that may be made by its manufacturer, is not guaranteed or endorsed by the publisher.

## Supplementary Material

Supplementary Figure S1 Pie chart fraction of *TERT* alterative subtypes occurring in *TERT*-alt HGMs.

Supplementary Figure S2 Heatmap of mutually exclusive or co-occurring set of genes in the mutation pattern of *de novo* HGMs following RT. Pairwise Fisher’s exact tests were performed to detect significant pairs of genes, mutually exclusive or co-occurring set of genes which colored by brown or green can be detected using the *somaticInteractions* function in R/Bioconductor package ‘maftools’.

Supplementary Figure S3 The *TERT*-alt status and progressive variables did not show any relevancy in progression group.
