# Supplementary material for: TERT Alterations Predict Tumor Progression in De Novo High-Grade Meningiomas Following Adjuvant Radiotherapy
Source: Front Oncol. 2021 Oct 29;11:747592. doi: 10.3389/fonc.2021.747592 (PMC8586415; doi:10.3389/fonc.2021.747592)
Supplement: Supplementary file 1 [file DataSheet_1.docx]

**Supplementary Material**

***Next-generation sequencing***

Twenty 4um thickness of formalin-fixed paraffin embedded (FFPE) tumor sections for each sample were used to extract DNA via HiPure FFPE DNA Kit (Magen, D3126-03) following the manufacturer’s protocol. DNA concentration was then measured using Qubit™ dsDNA HS Assay Kits (Thermo Fisher Scientific, Q32854) and the Qubit 3.0 fluorometer (Thermo Fisher Scientific) as described by the manufacturer. Genomic DNA libraries were constructed using KAPA Hyper Prep Kits (KAPA, KK8504) and captured using SureSelect Capture Library (Agilent, 772471) according to the manufacturer’s protocol. High-throughput sequencing was performed on Illumina miniseq platform.
