# Supplementary material for: TERT Alterations Predict Tumor Progression in De Novo High-Grade Meningiomas Following Adjuvant Radiotherapy
Source: Front Oncol. 2021 Oct 29;11:747592. doi: 10.3389/fonc.2021.747592 (PMC8586415; doi:10.3389/fonc.2021.747592)
Supplement: Supplementary file 2 [file Table_1.docx]

| **Age, mean ± SD** |  | 50.16±12.5 |
| --- | --- | --- |
| **Gender, n (%)** |  |  |
|  | Female | 135 (78.48) |
|  | Male | 37 (21.51) |
| **Localization, n (%)** |  |  |
|  | Non-skull base | 126 (73.26) |
|  | Skull base | 46 (26.74) |
| **WHO grade, n (%)** |  |  |
|  | II | 141 (81.97) |
|  | III | 31 (18.03) |
| **Histological subtype, n (%)** |  |  |
|  | Atypical | 140 (81.40) |
|  | Atypical/Chordoid | 1 (0.58) |
|  | Anaplastic | 30 (17.44) |
|  | Rhabdoid | 1 (0.58) |
| **Initial status, n (%)** |  |  |
|  | *De novo* | 51 (29.65) |
|  | Recurrent | 121 (70.35) |
| **Simpson grade, n (%)** |  |  |
|  | GTR | 148 (86.05) |
|  | STR | 24 (13.95) |
| **Postoperative radiotherapy, n (%)** |  | 87 (50.58) |

**Table S1.** Demographic characteristics of 172 patients with high-grade meningioma

GTR: gross total resection, STR: subtotal resection; n, number; SD, standard deviation.
