# Supplementary figures and images for: TERT Alterations Predict Tumor Progression in De Novo High-Grade Meningiomas Following Adjuvant Radiotherapy
Source: Front Oncol. 2021 Oct 29;11:747592. doi: 10.3389/fonc.2021.747592 (PMC8586415; doi:10.3389/fonc.2021.747592)

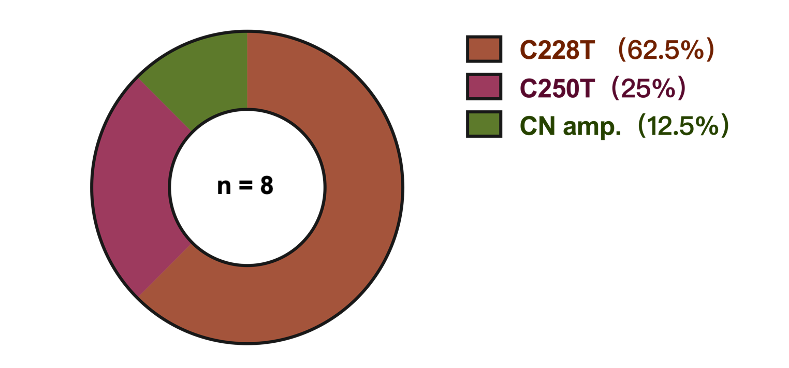

Supplement: Supplementary Figure S1 — Pie chart fraction of TERT alterative subtypes occurring in TERT-alt HGMs. [file Image_1.tiff]

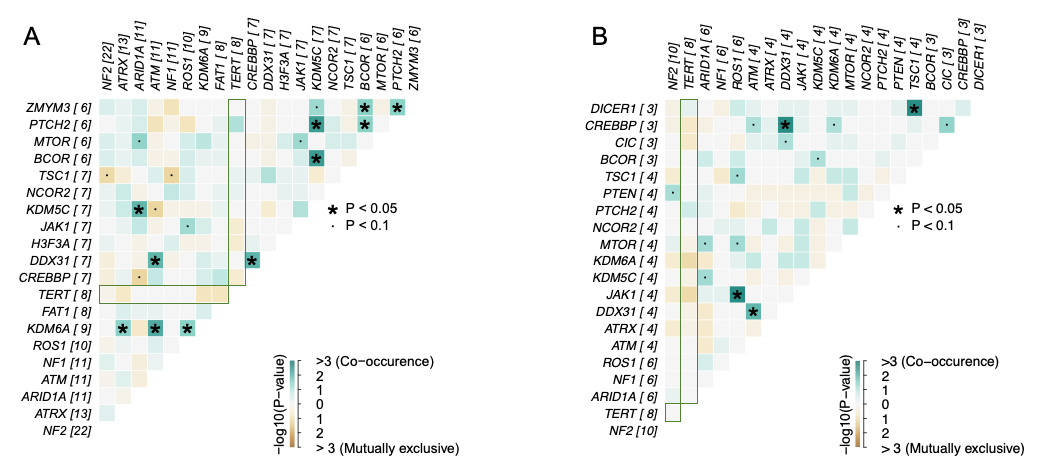

Supplement: Supplementary Figure S2 — Heatmap of mutually exclusive or co-occurring set of genes in the mutation pattern of de novo HGMs following RT. Pairwise Fisher’s exact tests were performed to detect significant pairs of genes, mutually exclusive or co-occurring set of genes which colored by brown or green can be detected using the somaticInteractions function in R/Bioconductor package ‘maftools’. [file Image_2.tiff]

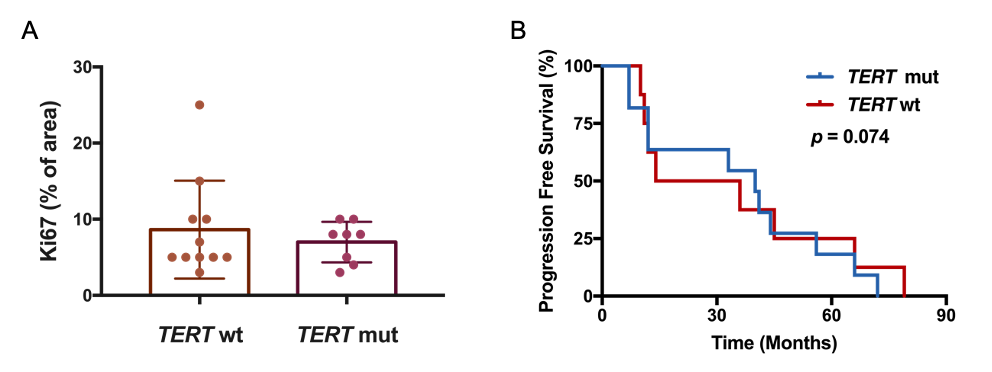

Supplement: Supplementary Figure S3 — The TERT-alt status and progressive variables did not show any relevancy in progression group. [file Image_3.tiff]
